# Supplementary material for: Soil microbiome perturbation impedes growth of Bouteloua curtipendula and increases relative abundance of soil microbial pathogens
Source: PLoS One. 2026 Jan 5;21(1):e0312218. doi: 10.1371/journal.pone.0312218 (PMC12768230; doi:10.1371/journal.pone.0312218)
Supplement: Attachment — Submitted filename: PLOS_Response to Reviewers.docx [file pone.0312218.s003.docx]

PONE-D-24-43622

Soil microbiome perturbation impedes growth of Bouteloua curtipendula and increases relative abundance of soil microbial pathogens

Dear Editor,

We appreciate the reviewers’ comments and have provided our rebuttal to each of the points below. In addition we provided a marked-up document that highlights each of the changes made.

Reviewer #1: The manuscript titled here “Soil microbiome perturbation impedes growth of Bouteloua curtipendula and increases relative abundance of soil microbial pathogens”. The MS provides significant insights into the impact of acute soil microbial community perturbations, such as autoclave sterilization, on the growth dynamics of Bouteloua curtipendula and subsequent microbial succession. The findings highlight critical interactions between soil bacteria, fungi, and plant growth, contributing to the broader understanding of microbial ecology and plant-microbe interactions in restored native habitats. The integration of genomic analysis with plant growth metrics strengthens the study's conclusions.
However, authors should address the following comments:
• Please avoid initiating the Introduction section with “ Although”. Consider beginning with a strong, context-setting statement that highlights the relevance of soil microbial perturbations in ecological studies or restoration efforts.

This has been updated

• MM section- “soil samples were diluted in distilled water” – please mention how much soil was taken in how much water.

500 mg of soil was diluted in 5 mL of water. This was mentioned elsewhere in the manuscript, but is now moved to the correct section.

• “1 g of soil on LB nutrient agar plates”- I g of soil was directly plated or suspended in some solution before plating. Please clarify.

The 1g of soil was plated directly onto the agar. This is now clarified in the MM section.

• The manuscript states that whole-genome sequencing was used for bacterial identification, whereas fungal identification relied on ITS region sequencing. Clarify why 16S rRNA gene sequencing was not employed for bacterial community analysis to maintain consistency in approaches. Discuss the rationale for choosing these methods.

16S rRNA sequencing focuses on identifying and quantifying bacterial species and community composition but does typically not provide information down to the strain level nor does it give metabolic insights, while whole genome sequencing (WGS) provides a more comprehensive view of the microbiome, including functional genes and potential for strain-level resolution and antimicrobial resistance detection. However when using a WGS approach, the fungal species are often underrepresented (due to the abundance of bacteria) and therefore a more targeted approach is beneficial. This reasoning is now added to the manuscript.

• Some sentences are long and can be broken into shorter for e.g. "In the non-sterilized samples, soil planted with B. curtipendula had a similar overall bacterial community to non-planted soil..." can be split to improve readability. Please check throughout the MS.

Several sentences have been restructured and split for clarity throughout the MS.

• Ensure consistent use of terms like "non-sterilized" vs. "not sterilized." Pick one term and stick with it throughout.

The term not-sterilized is now use throughout the MS consistently (including in the figures)

• Similarly, ensure terms like "planted soil" vs. "planted condition" are uniformly used

This is now updated and ‘Planted soil’ is used consistently.

• The distinct recovery timelines for bacterial (4 weeks) and fungal (7 weeks) communities are noteworthy. However, the study does not delve deeply into potential ecological mechanisms underpinning these differences. Could the bacterial recovery be attributed to faster replication rates or the presence of spore-forming taxa that survived sterilization? Conversely, do fungal communities rely more heavily on external inoculation or plant root-associated processes for recovery?

Yes, we had eluted to both of these points in the results and the discussions, but we now have expanded on these more in the discussion section.

• The observed transient increase in potential pathogenic bacteria (e.g., Acidovorax, Xanthomonas) following sterilization and their subsequent decline suggest competitive exclusion or suppression by other microbial taxa. The authors should expand on the mechanisms driving pathogen suppression.

We had mentioned earlier in the discussion that fungal growth (for example Epicoccum sp.) has been associated with production of antimicrobial compounds against phytopathogens. We have now also expanded on the importance of the bacterial diverse microbiome to create an environment that is less conducive for growth of pathogenic organisms in the discussion.

• Were any functional genes associated with PGPB activity (e.g., nitrogen fixation or siderophore production) detected in metagenomic analyses, even at low abundance?

Those genes are certainly present in the analysis, but the gene abundances are not significantly different, and since we didn’t measure actual expression levels we could not expand on any possible production level differences.

• The observation of similar metabolic pathway profiles across soil conditions, aside from sterile soils at time 0, suggests functional redundancy in the microbial community. Could this indicate that even after disruption, soil microbiomes can maintain core ecosystem services, albeit with altered taxa? Discussing this in the context of resilience and ecosystem functionality could strengthen the manuscript

We have expanded on this in the discussion.

• In some of the figures, the error bar is out of the scale. Please correct.

In some figures the error bars are approaching or reaching the outside frame. Therefore we removed the outside frame from the graphs in Figures 3 A-D. That shows the entire graph and error bars now.

Reviewer #2: Comments to the authors:

How did the study ensure that the observed differences in B. curtipendula growth were solely due to soil microbial communities rather than other abiotic factors altered by sterilization (e.g., nutrient availability, soil structure, or chemical changes)?

We did not test for all these factors and some of these can possibly impact the initial growth (of both microbes and plants). But if so, the plant growth rates are restored after microbes reestablish. If the sterilization changes additional properties of the soil, the plant growth inhibition of this change is ameliorated by the recolonization of bacteria/fungi. We have expanded on this briefly in the discussion section.

The study found that bacterial communities in sterilized soil eventually became similar to those in non-sterilized soil. What mechanisms or sources contributed to this microbial recolonization, and could this transition period impact long-term plant establishment and survival in prairie restorations?

In an open environmental system (even within a greenhouse setting) there is an abundance of environmental organisms that given the right conditions will propagate. Even minimal amounts of survival organisms in autoclaved soil can quickly recolonize. It is difficult to speculate in the current model to specify the source or mechanism of recolonization, which would require detailed follow up studies. The timing of the transition period and recolonization after an acute disturbance in an environmental setting would certainly impact the establishment of the plants. We have expanded upon this more in the discussion.

Given that urban grasslands and prairie restorations are the intended application areas, how do the study's findings translate to real-world settings with naturally occurring microbial heterogeneity and environmental stressors (e.g., drought, pollution, competition from invasive species)?
We have expanded upon this in the discussion and made comparisons between our simulated acute disturbance and the processes that occur during prairie restoration or urban native garden projects.

Is the method of microbial perturbation (autoclave sterilization) a reliable proxy for real-world soil disturbances in urban environments?

We have expanded upon this in the discussion and made comparisons between our simulated acute disturbance and the processes that occur during prairie restoration or urban native garden projects.

Are the high-throughput sequencing methods used in the study robust enough to capture the functional roles of microbial communities in pocket prairie soil?

The high-throughput sequencing methods used in this study are the current standard for metagenomics studies in complex microbiomes in many environments. Our studies did not address any specific functional roles of specific species or species interactions in these environments, which would require follow up transcriptomics or proteomics-based studies or follow-up growth experiments with specific bacterial or fungal species. These are great follow up studies, but currently fall beyond the scope of this paper.

How was it ensured that the sterilization process did not alter the soil’s physical and chemical properties beyond microbial depletion, potentially affecting plant growth independently of microbial presence?

This is possible. But if so, the plant growth rates are restored after microbes reestablish. If the sterilization changes additional properties of the soil, the plant growth inhibition of this change is ameliorated by the recolonization of bacteria/fungi. We have expanded on this briefly in the discussion section.

How were plant height and blade numbers recorded to minimize measurement error and observer bias? Were multiple measurements taken per plant, and was there any calibration or standardization of the measurement tools?

All measurements were performed by a single individual to ensure consistency over time. At the start of the study, measurements were compared between two individuals to conclude minimal impact on between-measurer variability. The calibration tools were standard supply rulers that did not require calibration. We have now included this in the MM section.

Was any quality assessment (e.g., Bioanalyzer, Qubit) performed before sequencing to ensure high-quality input DNA for WGS and ITS sequencing?

Yes, we performed Qubit analysis in addition to the Nanodrop measurements of the DNA and the prepared libraries before sequencing. This is now added to the Material and Methods.

Can the authors provide more details on the distribution of sequencing depth across different samples? Were there significant variations, and if so, how were they accounted for in downstream analyses?

There was some variation in the read counts per sample (ranges are provided in the paper) although all samples used in the analysis had sufficient minimal read coverage. MG-RAST uses DEseq for normalization, which we had indicated in the paper. DEseq is an R package used to analyze count data from high-throughput sequencing assays. DESeq, as it has been shown to outperform other methods of normalization, in particular those that use any sort of linear scaling.

Was any rarefaction analysis performed to ensure sufficient sequencing depth for comparative analyses?

Yes, rarefaction analysis was performed on both the MG-RAST and BaseSpace Metagenomics output, and all samples were found to be with sufficient sequencing depth for further analysis (with the exception of the one sample that generated low data as we explained in the text). We have now included a more detailed section in the Material and Methods about the rarefaction, normalization and other statistical analysis that was performed.

How does the accuracy of MG-RAST compare to other taxonomic classification tools such as Kraken2, Kaiju, or MetaPhlAn for metagenomic analysis?

MG-RAST is a well-established tool for reference-based classification and provides an online metagenomic analysis interface that includes data uploading, QC and alignment with reference databases. It has the option of a no command line use through the web-portal. The advantage of MG-RAST is that not only taxonomic classification is provided, but also functionals subsystem analysis as part of the comprehensive data analysis. This does lead to longer computing time necessary for aligning contigs to a reference. It is a highly accurate method but has the disadvantage of being slow. To speed up the process, alternative methods such as Kraken have been developed to replace the direct alignment of a query against a reference database by a fast-lookup method of fixed-length k-mers extracted from the query. Other studies have shown that Kraken 2 and MG-RAST generate comparable results and that a reliable high-level overview of sample is generated irrespective of the pipeline selected. As long as up to date databases are used for classification. Many analysis packages are now available and their accuracy and validity have been compared extensively in the last years. Here are some examples of publications that used MG-RAST in their comparisons to other bioinformatic resources:

<https://pubmed.ncbi.nlm.nih.gov/32382536/>

<https://www.sciencedirect.com/science/article/pii/S2001037021004931>

<https://pmc.ncbi.nlm.nih.gov/articles/PMC5861821/>

<https://pmc.ncbi.nlm.nih.gov/articles/PMC7641418/#R205>

Were any de novo assembly-based methods used alongside MG-RAST to improve species-level classification?

No

What were the specific reasons for the failed sequencing of the replica sample (sterile soil with B. curtipendula)? Could this indicate issues with DNA extraction or library preparation?

The DNA and library prep assessment looked fine, but resulted in low sequencing output. The sequencing of that sample was never repeated since we had 5 other valid replicas.

Were biological replicates used in addition to technical replicates to assess the reproducibility of taxonomic assignments?

All of the samples were taken from their individual growth experiment, so they are all biological replicates.

The study reports significant differences in the germination rates of B. curtipendula in sterilized versus non-sterilized soils. Could other abiotic factors, such as changes in nutrient availability or soil structure post-autoclaving, have influenced germination in addition to microbial community alterations?

As mentioned above, this is possible. But if so, the plant growth rates are restored after microbes reestablish. If the sterilization changes additional properties of the soil, the plant growth inhibition of this change is ameliorated by the recolonization of bacteria/fungi. We have expanded on this briefly in the discussion section.

The bacterial community in sterilized soils eventually converges towards that of non-sterilized soil by week 7. Was any quantitative measure (e.g., alpha or beta diversity indices) used to evaluate the pace of microbial succession?

The alpha diversity is shown in the Shannon index in Figure 3. The beta diversity is the change in community across sample conditions, which we show in Figure 2 ordination plots. Although we didn't quantitate microbial diversity changes, we did use Adonis multivariate ANOVA to statistically test the change in community composition across time points and conditions.

The study identifies shifts in metabolic pathways (e.g., carbohydrate metabolism, dormancy, and sporulation) following soil sterilization. Can these shifts be directly linked to changes in plant growth parameters?

That is a good point, but given the diversity and complexity of these metabolic pathways, a more detailed study of pathways and potential microbe-plant interactions would be required, which is beyond the scope of the current paper.

The study uses one-way ANOVA for comparing growth parameters. Were assumptions of normality and homogeneity of variance tested before applying ANOVA?

We did not test for homogeneity of variance.

The findings suggest that seed-associated fungi (Alternaria, Epicoccum) preferentially colonized sterile soil but not non-sterile soil. Could this be due to competitive exclusion in non-sterile soil, and was any additional sequencing of seed-associated microbes conducted to further explore this hypothesis?

This could indeed indicate a competitive exclusion of *Alternaria* and *Epicoccum* in the not-sterile soil by species that were already present from the start however further detailed studies on comparative growth rates and characterization of these species would be needed to clarify this. We have not conducted any follow up growth or sequencing studies to test this, however we have included this suggestion in the manuscript.

How did the researchers confirm that the initial bacterial and fungal communities were representative of typical prairie soil conditions? Could there be a potential bias introduced by the sterilization process that alters the baseline microbial community differently than anticipated?

The purpose of this study was not to study existing prairie soil, but merely to study the *Bouteloua* microbiome in a controlled environment. We have performed previous studies on comparing environmental soil microbiomes from native plant areas (that included *Bouteloua*) to turf grass areas, and we are currently performing a field study in native soil conditions where *Bouteloua* is introduced after a soil disturbance. The results from that study will be compared to the microbiomes established under greenhouse conditions.

How were the control groups structured, particularly regarding soil conditions? Were there any unsterilized soil groups where B. curtipendula was not seeded to isolate the effects of soil sterilization alone versus the interaction with the grass?

Yes, one of the control groups was not-sterilized and not-planted, as described in the manuscript.

The study suggests that Rhodopseudomonas and Mycothermus play roles in promoting plant growth. How were these roles verified experimentally, beyond correlation? Is there direct evidence linking the presence or absence of these microbes to the observed changes in B. curtipendula growth?

We did not follow up yet with specific experiments of these on B. curtipendula growth, however the plant promoting properties (on other plants) of these species have been shown in numerous publications and particularly for Rhodopseudomonas, have been exploited commercially. References to these studies are already included in the paper.

What mechanisms are proposed for the transient increase in potentially pathogenic bacterial genera like Acidovorax, Cellvibrio, and Xanthomonas in sterilized soils? Could other environmental factors, such as soil nutrient changes due to autoclaving, contribute to this shift?

We had mentioned earlier in the discussion that fungal growth (for example Epicoccum sp.) has been associated with production of antimicrobial compounds against phytopathogens. We have now also expanded on the importance of the bacterial diverse microbiome to create an environment that is less conducive for growth of pathogenic organisms in the discussion. Any factor that causes a dysbiosis in diverse microbiome (autoclaving, nutrient change, etc.) allows for opportunities of pathogens to increase.

How did the authors ensure the accuracy of the metagenomic sequencing data, particularly given the challenges of identifying microbes at the species level? Were there any potential biases in the sequencing or data analysis that might affect the results?

We included six replicas of each condition in our sequencing experiments to reduce any bias or eliminate potential sequencing outliers. In addition, we used standard methods for normalization and data rarefaction within MG-RAST as part of the data analysis.

The authors suggest that incorporating growth-promoting microbes into seeds could enhance the establishment of native grasses. How would this approach account for the complex and dynamic interactions between soil microbes and plant roots over time, especially in varied environmental conditions?

The suggestion of incorporating growth promoting microbes into seeds was proposed based on concepts that have been performed with crop plants (references are included). Such methods have not been tested with native grasses (to our knowledge), which is why this initial exploratory study was performed. The long-term effects on plant roots and exposure to varied environmental conditions would certainly have to be tested in future projects, however our initial study indicates that the same inoculation concepts could possibly provide benefit to the initial establishment of the native grass, which is often a crucial step in restoration projects. We have briefly expanded on this in the manuscript.
