# Supplementary material for: Soil microbiome perturbation impedes growth of Bouteloua curtipendula and increases relative abundance of soil microbial pathogens
Source: PLoS One. 2026 Jan 5;21(1):e0312218. doi: 10.1371/journal.pone.0312218 (PMC12768230; doi:10.1371/journal.pone.0312218)
Supplement: Attachment — Submitted filename: PLOSOne_revision2_Response to Reviewers.docx [file pone.0312218.s004.docx]

PONE-D-24-43622R1

Soil microbiome perturbation impedes growth of Bouteloua curtipendula and increases relative abundance of soil microbial pathogens

Dear Editor,

We appreciate the reviewers and your comments and have provided our rebuttal to each of the points below (in red). In addition, we provided a marked-up document that highlights each of the changes made.

Additional Editor Comments:

1. Material and Methods Section: Please provide proper citations for the materials, methods, instruments, and tools used in this study. For example, the tools used to measure temperature, humidity, and light levels should be cited appropriately. This will allow readers to better understand the methodology and replicate the study if needed.

Temperature, humidity and light levels were controlled using the Greenhouse Wadsworth control systems using aspirated temperature/humidity sensors and PAR light sensors. The specific aspirator sensor model number has been added to the manuscript.

2. Sample Collection Depth: For the soil collection process, please specify the depth at which the samples were collected. This detail is important for understanding the representativeness and the potential variability of the soil microbial communities across different soil layers.

We collected the entirety of the soil. For these experiments, seeds were sown in small plug trays. At collection points, the plant material was removed and the entire plug of remaining soil was homogenized and sampled. This information is now added to the Mat and Meth section.

3. EPA Method: In the material and methods section, please mention the specific Environmental Protection Agency (EPA) method or protocol followed for soil sample collection and analysis. This will ensure the method’s credibility and alignment with established standards for environmental and microbiological studies.

We did not follow a specific EPA method. The available EPA methods are specifically designed for field soil measurements and not for greenhouse plant/soil sampling. The protocol steps are explained in detail in the Mat&Meth section and should allow for reproducibility by the readers.

4. Seed Germination Measurement: How was seed germination measured in this study? Please specify the method used and provide the relevant reference for this procedure. Additionally, please elaborate on why the sterile soil showed a higher number of germinations compared to non-sterile soil. This is a crucial aspect to help readers understand the environmental conditions that influenced germination rates.

In order to allow longitudinal study without disturbing the soil between collection points and to pair-match soil samples with plant growth data, germination was inferred as visible cotyledon. This is now added to the Mat & Meth section of the manuscript. Although more sensitive or specific measures for germination could be useful, they lack the convenience and capability of analyzing undisturbed samples.

5. WGS Output and NMDS Analysis: In the results section, it is mentioned that WGS output was analyzed by selecting all reads aligned to the domain Bacteria, followed by NMDS analysis at the taxonomic level of genus. Could you please elaborate on this process? Specifically, how were the reads selected, and what was the rationale for performing NMDS analysis at the genus level? Additional details will help readers understand the methodology and the choices made during the analysis.

The genus level is the standard for microbiome comparisons. None of the current methods allow for enough resolution at the species level. We don’t have enough resolution at the species level to have confidence in the species metric, so the genus level NMDS analysis must suffice. The species-level NMDS could over or underestimate microbial community differences between samples due to misidentifying particular reads as a given species.

6. Figure 4 Clarifications: In Figure 4, the comparison between "sterile not planted" vs "sterile planted" shows higher bacterial and fungal diversity. Can you provide more details about this observation? Specifically, what could explain the higher diversity in the planted sterile soil?

We believe this was already discussed (this relates to Figure 3, not Figure 4) and the explanation is that the plants likely provided additional niches to support new bacterial and fungal taxa. There is also an increase in potential plant pathogens following the introduction of plants to the sterilized soil. We have briefly expanded on this with the Figure 3 diversity figure in the Result section.

7. The legend refers to Figure 4A and 4B, but these sub-figures are not included in the manuscript. Please check all figures and legends to ensure consistency and clarity.

The line item in the text referring to 4A and 4B has been corrected. This referred to a previous version of the figure.

8. The "soil metabolic pathways" shown in Figure 4 appear to be different from the figure referenced in the text. Kindly review and revise the figures and legends accordingly.

The names of the metabolic pathways in the figure 4 and in the text describing this section (lines 353-360) are identical, and all are present in the figure. We are not sure where a revision might be needed.

9. Figure Creation Process: Please specify in the methods section how the figures were created. Include the source of the input files, the file types used, and the tools/software utilized to generate the figures. For instance, it is unclear how Figure 5 was generated and how the relative abundance of soil metabolic pathway subsystems was calculated. Please clarify this process.

All the figures were generated from raw Illumina sequence processing as described in the materials and methods. Delim .txt files of processed sequence data were analyzed in R, as described in the materials and methods. The relative abundance was calculated simply as the proportion (or percentage) of reads identified as a particular taxonomic unit out of all of the total reads. Or, in the case of soil metabolic pathway subsystems, the relative abundance is the percentage of reads for a particular subsystem out of all the reads analyzed. All analyses were performed in R and all graphs were generated in R using ggplot2. This information is included in the Mat & Meth, however we could provide the specific R scripts if deemed necessary (maybe as supplemental?). Including the specific, basic R scripts within the manuscripts seems like it would be outside the scope of the paper.

10. Raw Data Submission: Kindly provide the raw data (Whole genome, 16S, ITS) used in this study in supplementary material. Additionally, submit these data to NCBI and provide the accession numbers for these datasets.

The data was already submitted to NCBI and the accession numbers are already in the manuscript: (lines 205-209) “Both the WGS metagenomic and targeted fungal sequencing datasets were deposited into NCBI Genbank under BioProject PRJNA1163419 and the WGS data are accessible with the following SRA numbers: SRR30751922-SRR30751939 and SRR30754582-SRR30754613. The targeted fungal ITS sequencing files are available with the following SRA numbers: SRR30786940-SRR30786954”
11. Figure 6 Clarification: In Figure 6, why were only ~30 phyla identified for bacteria and 7 for fungi at the phylum level? Please provide an explanation for this observation.

The threshold of phyla greater than 2% of the rarefied reads is already included in the figure description. The phyla below the threshold were pooled into ‘other’ as described in the legend.

12. Terminology Adjustment: Please modify the terminology from "whole genome" to "bacterial whole genome" or simply "bacteria" to provide a clearer indication of the focus on bacterial and fungal studies in your manuscript.

The whole genome sequencing approach was not limited to bacteria only and therefore changing the terminology would be misleading. In the previous revision it was clarified why we did additional fungal ITS sequencing in addition to the WGS.

13. Basidiomycota and Actinobacteria Observations (Fig. 7):In Figure 7, it is noted that Basidiomycota were present in week 4 and week 7, but not at week 0, and the same observation is made for Actinobacteria. Could you please explain the reason behind this temporal variation? Additional details on this pattern will help readers understand the ecological implications.

We have now expanded on this observation in the Discussion section. The lack of these at week 0 is clearly due to the sterilization. However as we indicated, the biochemical reason for a possible inhibitory effect on Actinobacteria and stimulatory effect of Basidiomycota due to planting will need further detailed follow up studies.

14. Alpha and Beta Diversity: Please provide the alpha and beta diversity metrics, both week wise and treatment-wise, to give a clearer understanding of the differences between the samples over time and under different conditions. This will help in interpreting the microbial diversity patterns in the study.

The diversity information is already provided (figure 3 and figure 2), and we described how Shannon and beta diversity were calculated in the Mat&Meth.


[Note: HTML markup is below. Please do not edit.]

Reviewers' comments:

Reviewer's Responses to Questions

**Comments to the Author**

1. If the authors have adequately addressed your comments raised in a previous round of review and you feel that this manuscript is now acceptable for publication, you may indicate that here to bypass the “Comments to the Author” section, enter your conflict of interest statement in the “Confidential to Editor” section, and submit your "Accept" recommendation.

Reviewer #1: All comments have been addressed

Reviewer #3: All comments have been addressed

2. Is the manuscript technically sound, and do the data support the conclusions?

The manuscript must describe a technically sound piece of scientific research with data that supports the conclusions. Experiments must have been conducted rigorously, with appropriate controls, replication, and sample sizes. The conclusions must be drawn appropriately based on the data presented.

Reviewer #1: Yes

Reviewer #3: Partly

3. Has the statistical analysis been performed appropriately and rigorously?

Reviewer #1: Yes

Reviewer #3: Yes

4. Have the authors made all data underlying the findings in their manuscript fully available?

The [PLOS Data policy](https://urldefense.com/v3/__http:/track.editorialmanager.com/CL0/http:*2F*2Fwww.plosone.org*2Fstatic*2Fpolicies.action*23sharing/1/010f0197d66cface-07ad4859-de0d-4e50-8948-b2025289a2fe-000000/-vNuz-XOcUCnCQ3bbI-pacAgkEHKYsbaH_YpQUjbyJY=218__;JSUlJSU!!NmoQVLtQOg!Aag6dj_VemzF8-Swc_oOn-j1Z_bx20iFapAGbOUI33NaC6DH7uKFDW_c4Iva5CgcTW_PB-4yE671Q2g87u8$) requires authors to make all data underlying the findings described in their manuscript fully available without restriction, with rare exception (please refer to the Data Availability Statement in the manuscript PDF file). The data should be provided as part of the manuscript or its supporting information, or deposited to a public repository. For example, in addition to summary statistics, the data points behind means, medians and variance measures should be available. If there are restrictions on publicly sharing data—e.g. participant privacy or use of data from a third party—those must be specified.

Reviewer #1: Yes

Reviewer #3: No

5. Is the manuscript presented in an intelligible fashion and written in standard English?

PLOS ONE does not copyedit accepted manuscripts, so the language in submitted articles must be clear, correct, and unambiguous. Any typographical or grammatical errors should be corrected at revision, so please note any specific errors here.

Reviewer #1: No

Reviewer #3: Yes

6. Review Comments to the Author

Please use the space provided to explain your answers to the questions above. You may also include additional comments for the author, including concerns about dual publication, research ethics, or publication ethics. (Please upload your review as an attachment if it exceeds 20,000 characters)

Reviewer #1: The manuscript titled here is “Soil microbiome perturbation impedes growth of Bouteloua curtipendula and increases relative abundance of soil microbial pathogens”.
The authors have addressed the comments from the previous reviewers. However, some typographical and formatting issues still remain.
• For example, the species name B. curtipendula is not italicized in the heading of the Results section, and appears underlined elsewhere. Please ensure consistent and proper formatting of scientific names throughout the manuscript.

The species was not italicized in the result section headers because those were italicized themselves and standard nomenclature would invert italicized words in such instances. Nevertheless, we italicized everything for clarity. We did correct the underlined species name in the Result section.

• Figure 1A: The proportions on the Y-axis appear to exceed the scale.

The bars reach the 1.0 value with is the top of the scale, but do not exceed it (there would not be a value greater than 1 in the proportion scale).

• I recommend a thorough proofreading of the entire manuscript to correct remaining typographical and formatting errors.

This has been performed by two of the authors and errors have been corrected where needed.

Reviewer #3: There are some critical issues that must be addressed before the manuscript can be considered for publication.

1. Experimental Design and Methodological Concerns
Lack of soil characterization: The study relies heavily on interpreting microbial community shifts, yet there is no physical or chemical analysis of soil properties beyond pH.

As mentioned in the first review round, we did not test for chemical analysis in these experiments. All experiments were performed with the same starting soil and our main focus was measuring the microbial community changes with planting and sterilization. Some of these factors could have possibly impact the initial growth (of both microbes and plants). But if so, the plant growth rates are restored after microbes reestablish. If the sterilization changes additional properties of the soil, the plant growth inhibition of this change is ameliorated by the recolonization of bacteria/fungi. We have already expanded on this briefly in the discussion section.

2. Language, Grammar, and Writing Style
Several long, multi-clause sentences would benefit from being split to improve clarity and reduce reader fatigue. There is occasional verb tense inconsistency, particularly between past and present tense in the Methods and Discussion sections.
Some stylistic redundancy exists in the Discussion, where results are repeated rather than critically analyzed.

The verb tense inconsistencies in the Math and Methods have been corrected (at least two occasions were found).

Due to the complex and multi-level comparisons of this study, we felt the need to repeat some of the results in some sections of the discussion for clarity to the reader. There are various sections where we discuss planted vs not planted, sterile vs not sterile, bacteria vs fungi, etc, and repeating a brief summary of the results seem necessary to keep clarity for the readers. We did change/expanded some of the discussion sections to put the emphasis more on analysis (or future studies) and rearranged small sections and broke up some of the discussion section, which will hopefully help the readers.

3. Formatting and Editorial Corrections
Capitalization: Please correct all instances of “illumina” to “Illumina”, as this is a proper noun referring to a company and sequencing platform.

The term Illumina occurs 14 times in the manuscript and is capitalized each time. However, in two of the figure legends, the term was autocorrected by Word to ‘illumine’, which has now been corrected to “Illumina”.

Figure Captions: Ensure all figure captions are written as complete sentences and include correct punctuation. For example:
Use proper spacing in statistical expressions (e.g., n = 6, not n=6).

Figure legends and statistical expressions have been updated.

7. PLOS authors have the option to publish the peer review history of their article ([what does this mean?](https://urldefense.com/v3/__https:/track.editorialmanager.com/CL0/https:*2F*2Fjournals.plos.org*2Fplosone*2Fs*2Feditorial-and-peer-review-process*23loc-peer-review-history/1/010f0197d66cface-07ad4859-de0d-4e50-8948-b2025289a2fe-000000/w5XhRjZEmwYed2inwMLFG1_7LEwM5JjHw6ArS-jPBr0=218__;JSUlJSUl!!NmoQVLtQOg!Aag6dj_VemzF8-Swc_oOn-j1Z_bx20iFapAGbOUI33NaC6DH7uKFDW_c4Iva5CgcTW_PB-4yE671aql41cc$)). If published, this will include your full peer review and any attached files.


If you choose “no”, your identity will remain anonymous but your review may still be made public.


**Do you want your identity to be public for this peer review?** For information about this choice, including consent withdrawal, please see our [Privacy Policy](https://urldefense.com/v3/__https:/track.editorialmanager.com/CL0/https:*2F*2Fwww.plos.org*2Fprivacy-policy/1/010f0197d66cface-07ad4859-de0d-4e50-8948-b2025289a2fe-000000/Y41OSJTlwxNXQhfXpkq9CBL3yHlwLb2qknheEBv2pdY=218__;JSUl!!NmoQVLtQOg!Aag6dj_VemzF8-Swc_oOn-j1Z_bx20iFapAGbOUI33NaC6DH7uKFDW_c4Iva5CgcTW_PB-4yE671onyDLX4$).

Reviewer #1: No

Reviewer #3: No
