# Supplementary material for: Soil microbiome perturbation impedes growth of Bouteloua curtipendula and increases relative abundance of soil microbial pathogens
Source: PLoS One. 2026 Jan 5;21(1):e0312218. doi: 10.1371/journal.pone.0312218 (PMC12768230; doi:10.1371/journal.pone.0312218)
Supplement: Attachment — Submitted filename: PLOSOne_Additional Editor Comments_R3.docx [file pone.0312218.s005.docx]

Bellevue, Sept 18, 2025

PONE-D-24-43622R2

Dear Editor,

We are glad that all the reviewer comments were satisfied and appreciate your additional comments for improvement. Below we provide our rebuttal to each of the points (in red) and pointed out which sections were further clarified in the text. In addition, we provided a marked-up document that highlights each of the changes made.

*Additional Editor Comments*
Dear Dr. Kyndt,

Thank you for the opportunity to review your manuscript, "Soil Microbiome Perturbation Impedes Growth of Bouteloua curtipendula and Increases Relative Abundance of Soil Microbial Pathogens." The study is valuable and well-written. I have a few minor suggestions:

1. Please provide proper research article citations for the methodologies described, such as material methods, plant growth measurements, soil pH measurements, LB agar plating, DNA extraction, next-generation sequencing, and data analysis (e.g., MG-RAST using DESeq, etc.).

The plant growth and soil pH measurement were not based on previous research articles and the methods and consumables used are described in this manuscript in a manner that should be reproducible by the reader. DNA extraction and NGS sequencing was performed using commercial kits and an in-house Illumina sequencer, and we described this in detail in the Mat and Meth. A reference for DESeq has been added in addition to the MG-RAST references that were already provided.
2. Please ensure that appropriate citations are provided for the instruments used in this study, including QuBit and NanoDrop.

The company and model information was added for both of these.
3. The authors mentioned performing whole genome sequencing (WGS) for bacteria and then separately using ITS primers. This needs clarification: Does the WGS approach imply shotgun metagenomics sequencing?

Yes, it is now added that WGS is a shotgun sequencing approach.
4. Could the authors clarify whether microbial cell viability tests were conducted for both sterile and non-sterile samples? Amplicon primers were synthesized by Sigma Aldrich, what does it means?

Viability tests were only performed to the sterile soil samples to identify any possible residual viable cultures (as described in the paper). Viability tests on all other samples would not be very informative as only a small percentage of the soil organisms would actually be able to be tested or cultivate under laboratory conditions, and hence this would not contribute anything to this study.

Sigma Aldrich is a well-known chemical company that synthesizes custom primer orders. They are now part of the larger Merck conglomerate. We indicated the Merck company, state and country references to this statement for clarity.
5. Please provide proper reference for your statement – which statement? I assume this refers to point 6 below…

6. While whole genome sequencing provides a comprehensive view of the microbiome, including functional genes and potential for strain-level resolution, however when using a WGS approach, the fungal species are often underrepresented (due to the abundance of bacteria) and therefore a more targeted approach is beneficial.

Two references were added here, and it was clarified that the lack of curated fungal sequences in the WGS databases are also a contributing factor.
7. How does the variation in sequencing depth (139,594 to 9,540,606 reads) impact microbial diversity representation, particularly for low-abundance species?

This is why rarefaction and normalization of the data was performed in the analysis (as indicated in the Mat and Meth section.) This point was also previously addressed in the reviewer comments.
8. How does sequencing depth influence community profiling and functional gene prediction? This can certainly have an impact, however all of our samples passed the MG-RAST initial QC that takes sequencing depth into consideration.
9. What strategies can mitigate fungal underrepresentation in WGS due to bacterial abundance? This is why we performed the additional targeted ITS sequencing on top of the WGS approach. This is explained in the text (and in comment 6 above).
10. Does the low fungal representation in WGS affect the interpretation of soil microbiomes and plant interactions? This is why we performed the additional targeted ITS sequencing on top of the WGS approach and the fungal results were discussed in conjunction with the bacterial results. Since we performed the targeted ITS fungal sequencing we can describe and compare the fungal impact. The ‘low representation’ is only a sequencing artifact and by taking the ITS fungal data into account we have provided a more realistic interpretation of soil microbes and plant interactions.
11. What biases might the Illumina Nextera DNA Flex Library Prep kit introduce in microbial taxonomic representation? The Illumina Nextera DNA Flex Library Prep kit was specifically designed to minimize the GC content and coverage biases that plagued its predecessor, the Nextera XT kit. However, no library preparation method is entirely free of bias. The most significant potential bias stems from the DNA extraction process, but the Flex kit itself can still introduce minor, though generally not substantial biases in taxonomic representation. 
12. How could these biases affect community structure and functional profile interpretation?

Multiple studies comparing Flex to other library prep methods found it introduces very little GC-related bias and produces a more uniform coverage across genomes with varying GC content. These are accepted standards and library preps in microbial genome sequencing and are the best we can currently do for analyzing community structure and functional profiling.
13. What quality and concentration thresholds were used in Qubit to assess library quality?

The purity and concentration levels for Qubit are added to the paper. All library samples had A260/280 ratios above 1.8 and all concentrations were above 10nM (which is the starting pool concentration).
14. How do these thresholds impact sequencing result accuracy and reproducibility?

All samples were well above these threshold.
15. How does Illumina MiniSeq's 2x150 bp read length affect strain-level resolution and functional annotation, particularly for complex soil microbiomes?

Any short read sequencing (and even long read) sequencing technique has issues with strain-level resolution in complex microbiomes, which is why we (and most other microbiome analysis papers) limit their comparison to genus (and sometimes species) level comparisons. Illumina’s 2x150 bp read length is a standard for microbiome level comparisons and well suited for genus level comparisons (not strain) and functional annotation. We are not performing strain level comparisons in this paper.

16. How does using the ITS1 region (18S to 5.8S rRNA genes) impact fungal taxonomic resolution in soil metagenomics? There is ongoing debate on which ITS region (ITS1/ITS2) is best for fungal genetic analysis, but both are widely used for metagenomic analysis. We chose the ITS1 region as per the demonstrated Illumina Fungal Metagenomic Sequencing Protocol (as indicated in the text).
17. Are there alternative markers that could improve fungal diversity analysis?

See answer to 16 above. The ITS regions are the only fungal markers that currently have sufficient database representation.
18. What measures can be taken to prevent or account for sample failures in sequencing and how do they affect result robustness?

Is this a general question? Every experiment will have to attempt to prevent sample failure and has a need for replicas. We have 6 biological replicas for each of the sampling conditions, which should provide sufficient robustness in the study. This is already described in the paper.
19. How do different sequencing cassette configurations (High-output vs. Mid-output) affect data quality and interpretation? The different cassettes do not impact data quality, the High-output simply allows for more samples per run, provided that there is enough sequencing depth for each sample.
20. How was data quality (Q30) ensured and what filtering criteria were used for consistency across the dataset?

The low quality data (<Q30) is already filter out by the sequencer data processing, however we performed an additional filtering of low qualtity scored reads using the FastQ Toolkit in BaseSpace (Illumina), as indicated under the Data Analysis Mat&Meth section.
21. What statistical methods assess the impact of sequencing variability (e.g., read numbers, data volume) on microbiome composition and functional interpretation?

As indicated in the text, rarefaction and normalization of the reads was performed either in R (scripts are now provided as supplemental info) or in MG-RAST itself.
22. Please include a section detailing the research contributions of each author to clarify their specific roles in the study.

A section with author contributions is now added after the Acknowledgements
23. Kindly provide a Declaration of Competing Interests to ensure transparency regarding any potential conflicts of interest.

A declaration of no conflicts of interest is now added after the Acknowldgements.
24. The legend for Figure 3 needs to be rewritten. It is currently unclear which diversity measure (alpha or beta diversity) is being discussed. Could you please specify and provide clarification?

It is now indicated in Figure 3 that alpha diversity (Shannon diversity index) is being discussed.
25. To enhance reproducibility, please provide the scripts or detailed procedure used for processing data with MG-RAST (or any other GUI tool). This should be included as a supplementary file so that other users can replicate your methods.

MG-RAST is a public service and scripts have been published before (and referenced in our paper). The additional R-scripts that we used and described in the manuscript are now submitted as supplemental information.
26. The legends for Figures 5, 8, and 9 require revision. The current descriptions are unclear. Additionally, please describe the figures as subfigures within the legend for clarity.

We have now restructured the descriptions for these figures to indicate more clearly what the subfigures are and how they should be interpreted, at the beginning of each description. We feel the methods and descriptions are clear as is but could address specific confusions if the editor has them.
27. Please correct writing style of phyla. Order, and genus in whole manuscript–
a) Phylum (or Phyla in plural) names are always capitalized and italicized or underlined. Example: Actinobacteria, Bacteroidetes
b) Order names are capitalized but not italicized. Example: Actinomycetales, Bacteroidales
c) Genus names are capitalized and italicized (or underlined if handwritten). Example: Streptomyces, Bacteroides
d) Species names are not capitalized but are italicized (or underlined if handwritten). Example: Streptomyces coelicolor, Bacteroides fragilis

The manuscript has been updated to reflect these recommendations.

Suggested Reference-
Caporaso JG, Lauber CL, Walters WA, Berg-Lyons D, Huntley J, Fierer N, et al. Ultrahigh-throughput microbial community analysis on the Illumina HiSeq and MiSeq platforms. The ISME journal. 2012;6(8):1621-4.

We are not sure why this paper is recommended. The paper discusses alternative Illumina platforms to the MiniSeq we used. Our MiniSeq instrument uses similar chemistry to the Nextseq technology, which is newer sequencing chemistry than the older Hiseq and MiSeq platforms. The newer platforms allow for faster sequencing and higher accuracy.

Pathak A, Jaswal R, Xu X, White JR, Edwards III B, Hunt J, et al. Characterization of bacterial and fungal assemblages from historically contaminated metalliferous soils using metagenomics coupled with diffusion chambers and microbial traps. Frontiers in Microbiology. 2020;11:1024

Although this is an interesting paper describing the bacterial and fungal communities in metal contaminated soils (particularly mercury) and an interesting DC/MT technology, since we did not examine metal content in our soil (and don’t suspect high metal contamination), we are not sure how this study is particularly relevant to our current analysis.

Gendy S, Chauhan A, Agarwal M, Pathak A, Rathore RS, Jaswal R. Is long-term heavy metal exposure driving carriage of antibiotic resistance in environmental opportunistic pathogens: a comprehensive phenomic and genomic assessment using Serratia sp. SRS-8-S2018. Frontiers in Microbiology. 2020;11:1923.

Similar to the reference before, this is an interesting paper in reference to long-term contaminated heavy metal habitats and the spread of antibiotic resistance by specific Serratia species in such sites. However since we did not find substantial levels of Serratia and have no indication of long-term heavy metal contamination in our soil (or in native prairie soils), adding this paper would create an additional justification and discussion that is beyond the scope of this paper.

Caporaso JG, Lauber CL, Walters WA, Berg-Lyons D, Lozupone CA, Turnbaugh PJ, et al. Global patterns of 16S rRNA diversity at a depth of millions of sequences per sample. Proceedings of the national academy of sciences. 2011;108(supplement_1):4516-22.

We are not sure why this reference is recommended. We did not perform 16S metagenomics analysis for our study. We purposely performed WGS metagenomics and have much higher coverage per sample than what is discussed in this (older) paper.

Clagnan E, Costanzo M, Visca A, Di Gregorio L, Tabacchioni S, Colantoni E, et al. Culturomics-and metagenomics-based insights into the soil microbiome preservation and application for sustainable agriculture. Frontiers in Microbiology. 2024;15:1473666.

This is a good general paper about holistic approaches for sustainable agriculture. Similar approaches could be used as we suggested in our discussion of native plant establishment. We added this reference (and another related one) and expanded briefly on this in our final discussion.

Caporaso JG, Lauber CL, Walters WA, Berg-Lyons D, Huntley J, Fierer N, et al. Ultrahigh-throughput microbial community analysis on the Illumina HiSeq and MiSeq platforms. The ISME journal. 2012;6(8):1621-4.

This is the same reference as the first one. See comment above.

Yang C, Mai J, Cao X, Burberry A, Cominelli F, Zhang L. ggpicrust2: an R package for PICRUSt2 predicted functional profile analysis and visualization. Bioinformatics. 2023;39(8):btad470.

We are not sure why this reference is recommended. We did not use this specific R package in our analysis.
